# Composing Optimized Embedded Software Architectures for Physics-Based EKF-MPC Smart Sensor for Li-Ion Battery Cell Management

**DOI:** 10.3390/s22176438

**Published:** 2022-08-26

**Authors:** Anne K. Madsen, Darshika G. Perera

**Affiliations:** Department of Electrical and Computer Engineering, University of Colorado Colorado Springs, 1420 Austin Bluffs Parkway, Colorado Springs, CO 80918, USA

**Keywords:** embedded software architecture, model predictive control, physics-based model, smart sensors, embedded systems, battery cell management

## Abstract

Efficient battery technology is imperative for the adoption of clean energy automotive solutions. In addition, efficient battery technology extends the useful life of the battery as well as provides improved performance to fossil fuel technology. Model predictive control (MPC) is an effective way to operate battery management systems (BMS) at their maximum capability, while maintaining the safety requirements. Using the physics-based model (PBM) of the battery allows the control system to operate on the chemical and physical process of the battery. Since these processes are internal to the battery and are physically unobservable, the extended Kalman filter (EKF) serves as a virtual observer that can monitor the physical and chemical properties that are otherwise unobservable. These three methods (i.e., PBM, EKF, and MPC) together can prolong the useful life of the battery, especially for Li-ion batteries. This capability is not limited to the automotive industry: any real-world smart application can benefit from a portable/mobile efficient BMS, compelling these systems to be executed on resource-constrained embedded devices. Furthermore, the intrinsic adaptive control process of the PBM is uniquely suited for smart systems and smart technology. However, the sheer computational complexity of PBM for MPC and EKF prevents it from being realized on highly constrained embedded devices. In this research work, we introduce a novel, unique, and efficient embedded software architecture for a PB-EKF-MPC smart sensor for BMS, specifically on embedded devices, by addressing the computational complexity of PBM. Our proposed embedded software architecture is created in such a way to be executed on a 32-bit embedded microprocessor running at 100 MHz with a limited memory of 128 KB, and still obtains an average execution time of 4.8 ms.

## 1. Introduction

Clean and renewable energy sources are critical for maintaining a livable environment and enabling energy independence. The economic shutdown due to the COVID-19 pandemic has demonstrated that reducing harmful emissions will result in a cleaner and healthier environment. The world-wide quarantine at the beginning of this pandemic has led to the reduction of harmful gasses such as nitrous oxide (NO_2_) by about 30% [1]. Although quarantine is not a long-term viable option, this illustrates what we could achieve by adapting clean energy solutions. Additionally, developing alternative energy sources enables countries to reduce reliance on energy imports, reducing the impact of foreign instability. For wide-scale utilization of clean energy, the overarching goal should be to make clean energy a viable, efficient, and cost-effective option that maintains or exceeds the current performance expectations. To facilitate this endeavor, we focus on a battery management system (BMS) that extends the useful life of the battery by preventing a known aging electrochemical reaction. Our intention is to create a smart sensor, based on a model predictive control (MPC), that controls and constrains the operations of the individual battery cells to prevent aging and prolong the useful life of the battery cell.

In order to reduce the computational load on the battery cell management, while retaining the ability to protect the battery cells from degradation of performance and extend their life, a modular BMS was proposed in [2,3] that enabled the control burden to be split among the subsystems. For instance, the DC–DC converter subsystem could handle the state-of-charge (SOC) balancing of the battery cells and the bus voltage regulation for powering the auxiliary systems, whereas a smart sensor based on a model predictive control (MPC) could be in charge of safe and reliable performance of individual battery cells.

Model predictive control (MPC) is an adaptive control approach that uses a model of a system to determine the optimal control needed to achieve an objective. In particular, MPC utilizes a “look-ahead” strategy to predict how the control signal would impact the system-under-control, and accordingly optimizes the signal based on the model and the constraints. One of the most effective techniques for cell-level monitoring and controlling of battery packs [4], MPC is designed to provide optimal performance while remaining within safety constraints. Typically, for the batteries, MPC depends on two main mathematical models: the equivalent circuit model (ECM) and the physics-based model (PBM). The ECM has the advantage of simplicity (i.e., comprises simplified algorithms) and dependable performance, but is limited in operating conditions and is unable to consider the electrochemical reactions inside the battery that lead to aging and other degradation. Conversely, the PBM is computationally complex, but is inherently adaptable to a wide range of operating conditions, and considers electrochemical reactions, enabling the MPC to consider aging, which in turn could prolong the useful life of the battery cells and enhance performance.

Many real-world applications/scenarios, such as hybrid electric vehicles, require portable battery management system (BMS), compelling BMS to be executed on resource-constrained embedded devices. However, the sheer computational complexity of PBM for MPC prevents it from being executed on highly constrained embedded devices, making it infeasible for portable BMS. The existing physics-based (PB) MPC designs, which often require significant processing power, are currently often realized on large computing systems. In addition, the intrinsic adaptive control process of the PBM is uniquely suited for smart systems and smart technology. Creating a portable (or mobile) PB-MPC would enhance the smart capabilities of the overall embedded systems. Hence, the challenge is to realize this vastly compute-intensive PB-MPC on resource-constrained embedded devices.

In this research work, we introduce a novel, unique, and efficient embedded software architecture for a physics-based (PB) MPC smart sensor for lithium-ion battery cell control. Since the electrochemical reactions are not physically measurable in a normal battery, the smart sensor utilizes an extended Kalman filter (EKF) as an observer to determine the internal states. In this paper, both the MPC and EKF use PBM; thus, our proposed solution is called a PB-EKF-MPC smart sensor. Moreover, both the PB-MPC and PB-EKF use state-space equations with overlapping functions, which in turn reduces the computation workload on embedded systems.

We investigated the existing works on PB-MPC in the published literature, and analyzed whether these designs are suitable for embedded devices. These investigations and analyses are presented in Section 4.3. Although there were a few designs for PB-MPC with EKF, most of these designs were executed on desktop computers [5,6,7] and most focused on Matlab modeling to verify and improve the physics-based (PB) approaches. Our work is inspired by the PB-MPC with PB-EKF proposed in [2,3], which is a Matlab model (executed on a desktop computer) that is focused on reducing the computation workload and increasing the accuracy and effectiveness of the battery models, which in turn could potentially facilitate realizing the PB-EKF-MPC on embedded devices with their stringent constraints/requirements.

From our investigation on the existing works (in Section 4.3), and to the best of our knowledge, no similar work exists in the published literature that provides embedded software architectures for a combination of EKF and MPC, specifically for physics-based (PB) battery management systems on an embedded microprocessor with a very limited memory of 128 KB.

In this paper, our contributions are as follows:

We introduce a novel, unique, and efficient embedded software architecture for a PB-EKF-MPC smart sensor for lithium-ion battery cell control. Our embedded software architecture is created in such a way to be lean in order to be executed on 32-bit embedded microprocessors with very limited memory of 128 KB.

Our proposed embedded software architecture can be utilized to control multiple battery cells individually, thus reducing the hardware resources required for BMS, which is ideal for resource-constrained embedded devices.

We perform experiments to evaluate the feasibility and efficiency of our embedded software design, which demonstrates that our proposed design for the PB-EKF-MPC smart sensor is indeed a viable approach for battery cell management.

The organization of the paper is as follows:

In Section 2, we present a brief description of the PB-EKF-MPC algorithm utilized for this research work, including the physics-based model (PBM), extended Kalman filter (EKF), and model predictive control (MPC). In Section 3, we present our proposed embedded software architecture for PB-EKF-MPC, which comprises the lean code structures utilized for our embedded software designs. In Section 4, we report our experimental results and analyses for our embedded software architecture, including the functional verifications with the baseline Matlab model, performance analysis, etc. Our analysis on existing works on embedded software designs for PB-EKF-MPC (in the published literature) is also discussed and presented in Section 4. In Section 5, we summarize our work, conclude, and discuss future directions.

## 2. Background: PBM, EKF, and MPC

In this section, we discuss and present the Physics-Based Model (PBM), Extended Kalman Filter (EKF) algorithm, and Model Predictive Control (MPC) algorithm utilized to create our unique and efficient embedded software architecture for the PB-EKF-MPC smart sensor.

### 2.1. Physics-Based Model (PBM)

There were several research works that focused on developing a simplified physics-based model (PBM), which strived to retain the benefits of adaptability and knowledge of electrochemical reactions to enhance the performance and life of batteries by preventing conditions that led to their degradation.

One early approach utilized a single particle model (SPM) to create each electrode as a single particle, while neglecting the electrolyte dynamics. The SPMe was an extension of SPM, which incorporated electrolyte dynamics back into the SPM [5], whereas others incorporated thermal effects [8,9].

The approach in this research work/paper utilizes a reduced order model (ROM) of a lithium-ion battery cell based on the work in [6,10,11]. We decided to focus on this approach, since it was stated in [6,10,11] that ROM was deemed especially suitable for embedded applications/devices, based on first order principles. In this approach, state-space representations are created using the Discrete Realization Algorithm (DRA) developed in [12] using a set points of temperature and state of charge (SOC).

In [2], the authors strived to reduce the complexity by combining two models of different temperature set points and the same SOC, in order to widen the accuracy of the resulting model while reducing the amount of stored data required for the application. This combined (or blended) model is the basis of the state and output matrices utilized in both the PB-MPC and PB-EKF algorithms as well as the non-linear corrections detailed in PB-EKF in Section 3.1. The baseline Matlab model for the PB-EKF-MPC is detailed in [2].

**Figure 1:**
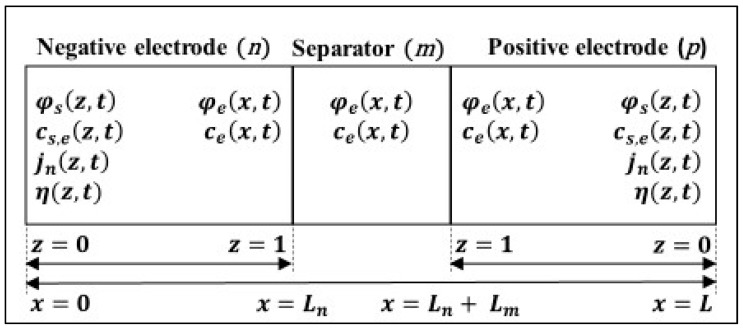
Three region cell model illustrating variables and their positions [12].

The analysis in [12] demonstrated that the PBM using five states is the best-case scenario considering the accuracy, performance, and complexity tradeoffs. Figure 1 illustrates the reactions that the PBM centers around. In this case, the minimum reactions required for the five states are the concentration in the electrolyte ($c_{e}$), solid surface concentration ($c_{s}$), total potential in the electrolyte ($\varphi_{e}$), electric potential in the solid ($\varphi_{s}$), flux ($j_{n}$), and overpotential (η). The side-reaction overpotential ($\varphi_{s-e}$) is an additional reaction used in BMS to regulate or prevent lithium plating, which is positioned in the PBM similarly to $\varphi_{s}$.

### 2.2. Extended Kalman Filter (EKF) Observer

In this research work, the observer is the Extended Kalman Filter (EKF) [2], which was developed based on the work in [6,10,11] using a PB-ROM that depends on the five basic reactions: $c_{s}$, $\varphi_{e}$, $\varphi_{s}$, $j_{n}$, and η. The EKF is employed to observe the non-measurable states that describe the internal electrochemical reactions of the battery. Although the observer is often utilized to monitor the SOC for the MPC, in this work, the SOC balancing is accomplished in the DC–DC converter. Moreover, in this work, our smart sensor is being employed to reduce the aging caused by lithium plating; thus, the EKF monitors the model states and the side reaction overpotential ($\varphi_{s-e}$), which is a major factor in the reduction of battery life and degradation in performance.

EKF is a non-linear adaptation of the linear KF that is ubiquitous in many engineering disciplines. The DRA method typically produces a completely linear model; hence, modifications are necessary to recover the non-linear behavior. As a result, the EKF used in this work follows the linear KF method, coupled with non-linear corrections required to use the state-space PBM. A detailed description of this PB-EKF can be found in [2] and it is also discussed in Section 3.1.

### 2.3. Model Predictive Control (MPC)

The PB-MPC algorithm follows the standard MPC flow. However, the PB-MPC utilizes the horizon-points method to reduce the prediction horizon, which in turn reduces the computational complexity while retaining stability [3]. For the PB-MPC, the current state is the observed state $x_{k+1}$ from the PB-EKF. Due to the integral action to the state-space equation, the PB-MPC operates on the difference between the current-state and next-state (i.e., $\Delta x_{m,k}$) as in Equation (1), whereas the PB-EKF operates on the state.

(1) $$\Delta x_{m,k}=x_{m,k+1}-x_{m,k}$$

The above parameter $x_{m,k}$ is the observed state output from the PB-EKF. In this case, by updating the state and output matrices in the final EFK computation, the state-space equations utilized for PB-EKF can be reused for PB-MPC.

In this work, the PB-MPC is a single-input/output approach that evaluates the cell current. When the cell current is within acceptable operating conditions, the PB-MPC acts as a pass through. If the cell current would cause lithium plating (a known aging mechanism), then the PB-MPC intervenes to take appropriate action. For this PB-MPC, the primary constraint is based on the side reaction overpotential ($\varphi_{s-e}$), which is a major factor in reduction of battery life and degradation in performance. A detailed description of this PB-MPC can be found in [2] and it is also discussed in Section 3.2.

## 3. Proposed Embedded Software Architecture for Physics-Based EKF-MPC

In this section, we discuss and present our novel, unique and efficient embedded software architecture for the physics-based (PB) MPC for the smart sensor. We initially investigate the functional flow of the PB-MPC [2,3], in order to gain insight, and then decompose this complex algorithm into several sub-tasks. This enables us to significantly simplify the design process without compromising the integrity and the performance of the baseline PB-EKF-MPC algorithm, which is imperative to realize this highly complex PB-EKF-MPC algorithm on resource-constrained embedded microprocessors.

Due to the limited resources of embedded microprocessors, it is crucial to reduce the code-size of our proposed embedded software architecture. Hence, we dramatically modified the baseline software designs that have been executed on desktop computer, so that they would fit into the highly constrained embedded microprocessor. In this case, we created the codes to be leaner and simpler in such a way as to fit into the available program memory of the embedded microprocessor, without impacting the basic structure or the functionalities of the PB-EKF-MPC algorithm. Many design decisions for software optimization involved reducing the computation workload of the original baseline software design which in turn facilitated the creation of lean and simpler codes for our proposed embedded software architecture. These design decisions and the corresponding efficient algorithm/code creations are detailed throughout this section.

**Figure 2:**
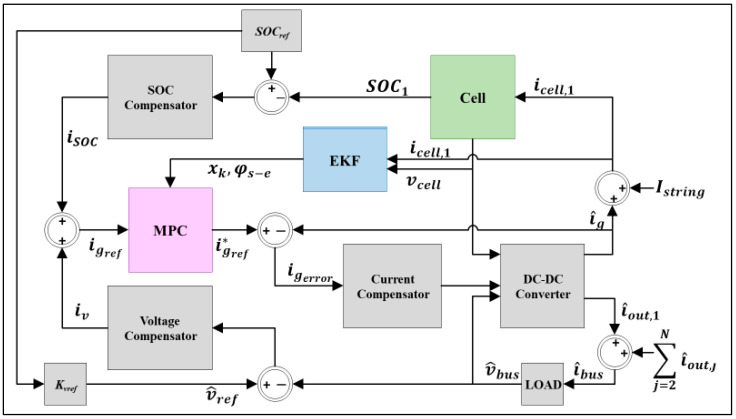
Block diagram of the BMS utilizing PB-EKF-MPC for smart sensor.

Figure 2 illustrates the block diagram of the BMS (battery management systems) that employs the PB-EKF-MPC. This Figure 2 is modified from [3] to comprise EKF and related functions. As depicted, the inputs to the EKF are the cell current (*i_cell_*_,1_) and the cell voltage (*v_cell_*), whereas the outputs of the EKF (i.e., the current-state of the battery, state of *φ_s-e_*, and state and output matrices (*A*, *B*, $\hat{C}$,$\;\hat{D}$)) are the inputs to the PB-MPC. In this case, the cell current is a combination of string current (*I_string_*) and control current (${\hat{i}}_{g}$). 

Since PB-MPC incorporates integral action, PB-MPC operates on the change-in (Δ) state from the current-state to the next-state and computes the change-in (Δ) current, whereas the EKF operates on the current-state. This change-in (Δ) current is added to the control current to produce a change-in (Δ) state for the PB-MPC with the aid of EKF. Then, the PB-MPC outputs an updated control reference signal ($i_{gref}^{*}$), which meets the constraints and *φ_s-e_* to prevent aging and battery degradation, while satisfying the required performance. In this work, both MPC and EKF use a physics-based model, and can thus be called PB-MPC and PB-EKF.

### 3.1. EKF

**Figure 3:**
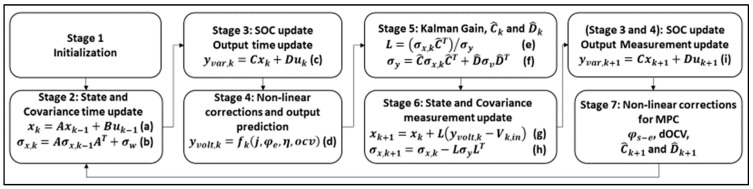
Software flow for PB-EKF.

The software functional flow of the PB-EKF algorithm comprises seven stages, and is shown in Figure 3, along with Equations (a)–(i) utilized for various stages. In Stage 1, the EKF is initialized with a state of charge (SOC) of 0.6 and *N_s_* is the number of states. With the EKF, the SOC is computed from the current state and the state update, where initial value serves as the current state of the SOC computation. Since the SOC cannot be physically measured, the initial value is often set to be 40–60% charged, to reduce the time taken for the observer to compute the correct SOC value [2]. In this case, the initial value of 60% = 0.6 performs well for the driving profile [13] utilized for our experiments in Section 4. 

In Stage 2, the EKF computes the state matrix (*A*) (in Equation (a) in Figure 3). The state matrix (*A*) is built using look-up-tables (LUTs) and 2D-interpolation based on the temperature and SOC values. The *A* matrix is computed using the following Equation (2):

(2) $$A\left[i\right]=\left(1-\alpha \right)\left(1-\beta \right)BigA_{0}\left[Low\right]\left[i\right]+\alpha \beta BigA_{1}\left[High\right]\left[i\right]\;\;+\left(1-\alpha \right)\beta BigA_{0}\left[High\right]\left[i\right]+\alpha \left(1-\beta \right)BigA_{1}\left[Low\right]\left[i\right]$$

In Equation (2), the α and β are the weight values based on the temperature and SOC, respectively; Low and High are the SOC floating point values; and *i* is the state. The original LUT for *A* matrix (*BigA*) was a 3D matrix, composed of two temperature values (0 and 25 °C), 20 integer representation of state of charge (SOC) values, and system state values (from 0–4 for the five states), which led to a *2X20X5* matrix. For our software design, we split the LUT into two *20X5* matrices (*BigA*_0_ for 0 °C and *BigA*_1_ for 25 °C). By rearranging the large 3D LUT into two smaller LUTs, we made it easier for our embedded software to store the matrices and reduce the number of LUT calls. The LUTs are further arranged by integer SOC values, derived from linear interpolation. The “High” and “Low” values bound the decimal value and are contiguously located in the LUT. In Equation (2), High and Low are fixed single values, where the decimal values are determined by the weights, α and β, respectively. Hence, the final state (*A*) matrix size depends only on the number of states (*i*). Since there are only five states, the final state (*A*) matrix becomes a *5X5* matrix. The diagonal nature of the state matrix allows *A* to be stored as a vector of diagonal values *A[i]*. The computation workload and the storage requirements are reduced by modifying and storing the *A* matrix as a five-element vector. The matrix (*B*) (in Equation (a), Figure 3) is a constant vector comprising values of all ones. 

For the PB-EKF, the control signal (*i_cell_*) is denoted by *u_k_*, state is denoted by *x_k_*, and the error covariance and the uncertainty of the current sensors are denoted by σ_x,k_ and σ_w_, respectively. Once the state matrices are computed, the PB-EKF performs the state (*x_k_*), (i.e., Equation (3) corresponding to Equation (a) in Figure 3) and the error covariance time update (σ_x,k)_, (i.e., Equation (4) corresponding to Equation (b) in Figure 3) based on the previous state, control signal, and error covariance matrix, using the following Algorithm 1:

(3) $$x_{k}=Ax_{k-1}+Bu_{k-1}$$

(4) $$\sigma_{x,k}=A\sigma_{x,k-1}A^{T}+\sigma_{w}$$

**Algorithm 1: Compute state and error covariance time update**$for\;i=1\;to\;N_{s}$ $\left\{x_{ekf}\left[i\right]=A\left[i\right]*x_{ekf}\left[i\right]+u_{k-1}\right\}$ $for\;i=1\;to\;N_{s}$ $\{for\;j=1\;to\;N_{s}$ $\left\{\sigma_{x}\left[i\right]\left[j\right]=A\left[i\right]*A\left[j\right]*\sigma_{x}\left[i\right]\left[j\right]+\sigma_{w}\left[i\right]\left[j\right]\}\right\}$

In Stage 3, in order to compute the output prediction, the PB-EKF initially estimates the SOC based on the state time update (*x_k_*), and the lithium concentration in the solid surface at the negative electrode ($c_{s}^{neg}$). For our software design, we use the cell model parameters from the Doyle cell parameters in [2]. These parameters are held constant for the temperature range (i.e., 0 to 25 °C) used for our software design, allowing the combination of constants and reducing the SOC computation to a linear equation. As a result, our output prediction computation is performed using Algorithm 2. Investigation and discussion on how these parameters would change with varying temperature ranges is beyond the scope of our work, but can be found in [2].**Algorithm 2: Compute SOC and lithium concentration based on the state time update**$c_{s,avg}^{neg}=m_{avg}*x_{ekf}\left[N_{s}-1\right]+b_{avg}$ $if\;c_{s,avg}^{neg}<0\;then\;c_{s,avg}^{neg}$ $if\;c_{s,avg}^{neg}>c_{s,max}^{neg}then\;c_{s,avg}^{neg}=c_{s,max}^{neg}$ $\theta^{neg}=c_{s,avg}^{neg}/c_{s,max}^{neg}$ $SOC=m_{SOC}\theta^{neg}+b_{SOC}$

Next step of PB-EKF (in Stage 3) is to compute the standard output function (*y_var,k_*) using Equation (5) (corresponding to the Equation (c) in Figure 3). In this case, the output matrices (*C* and *D*), derived from the DRA process (as in [12]), are entirely linear; thus, EKF requires extra steps to compute the non-linear output predictions. The *C* and *D* matrices are computed using LUTs and 2D interpolation, similar to *A* matrix; however, the internal organization of LUTs are different. The weight values α and β and input indices are updated based on the new SOC value. The size of matrix *C* is equal to *number-of-states* multiplied by *number-of-outputs*, where the outputs depend on the number of electrochemical reactions. In this research work, C is a *5X19* matrix. The *D* matrix is a vector, where length is equal to *number-of-outputs*, i.e., 19 in this work. These 19 outputs comprise non-linear correction values, which include in sequence: 4 values for concentration in the electrolyte ($c_{e}$); 3 values for potential in the electrolyte ($\varphi_{e}$); 4 values for the solid surface concentration ($c_{s}$); 4 values for the electric potential in the solid ($\varphi_{s}$); and 4 values for the flux ($j_{n}$). Our output function is computed using Algorithm 3:

(5) $$y_{var,k}=Cx_{k}+Du_{k}$$

**Algorithm 3: Compute the standard output function**, *y_var,k_*$for\;i=1\;to\;N_{out}$ $\left\{Clear\;C_{x_{ekf}}\left[i\right]\right\}$ $for\;i=1\;to\;N_{out}$ $\{for\;j=1\;to\;N_{out}$ $\left\{C_{x_{ekf}}\left[i\right]=C\left[i\right]\left[j\right]*x_{ekf}\left[j\right]\;\right\}\}$ $for\;i=1\;to\;N_{out}$ $\left\{y_{var}\left[i\right]=C_{x_{ekf}}\left[i\right]+u_{k}*D\left[i\right]\right\}$

As depicted in Figure 3, each element of *y_var,k_* represents the debiased value of an electrochemical reaction at a given position. The debiased value does not represent a physical reaction until non-linear corrections are performed. Hence, in Stage 4, once the non-linear corrections are performed on the five states (as detailed in Figure 3), the output voltage (*y_volt,k_*) is computed using Equation (6) as follows:

(6) $$y_{volt,k}=y_{var,k}\left[17\right]FR_{film+}-y_{var,k}\left[15\right]FR_{film-}+\varphi_{e}+\Delta \eta -u_{k}R_{term}+\Delta OCV$$

The above parameters, $FR_{film,pos}$, $FR_{film,neg}$, and $R_{terminal}$, are the resistances in the film multiplied by Faraday’s constant and the cell series resistance, and are pre-computed constants, applicable to the temperature range and SOC for the physics based (PB) model [2]. The terms $\;\varphi_{e}$ and Δη are outcomes of the non-linear corrections, and are computed using the following Equations (7) and (8).

(7) $$\varphi_{e}=y_{k}\left[6\right]+T_{k}\varphi_{const}\ln\frac{c_{e,L}}{c_{e,0}}$$

(8) $$\Delta \eta =\eta_{L}-\eta_{0}=\frac{T_{k}2R}{F}\cdot \left(\mathrm{asinh}\left(\frac{y_{k}\left[17\right]}{j_{0,\;pos}}\right)-\;\mathrm{asinh}\left(\frac{y_{k}\left[15\right]}{j_{0,\;neg}}\right)\right)$$

where, $c_{e,0}=y_{var,k}\left[0\right]+1$, $c_{e,L}=y_{var,k}\left[3\right]+1$, $c_{s,0}=y_{var,k}\left[7\right]+b_{c,neg}$, $c_{s,L}=y_{var,k}\left[7\right]+b_{c,pos}$, $j_{0,neg}=\sqrt{\left(\left(c_{s,\;neg,\;max}-c_{s,0}\right)c_{s,0}\cdot c_{e,0}\right)}\cdot e^{\left(-m_{e,neg}\cdot \frac{1}{T_{k}}+b_{e,neg}\right)}$, $j_{0,pos}=\sqrt{\left(\left(c_{s,\;pos,\;max}-c_{s,L}\right)c_{s,L}\cdot c_{e,L}\right)}\cdot e^{\left(-m_{e,pos}\cdot \frac{1}{T_{k}}+b_{e,pos}\right)}$.

The final term (ΔOCV) in Equation (6), is the open circuit voltage correction based on the SOC. This is also computed using the LUTs, linear interpolation, and weight factor.

In Stage 5, since the output equation (*y_volt,k_*) (i.e., Equation (9) corresponding to the Equation (d) in Figure 3) is non-linear, new linearized *C* and *D* matrices ($\hat{C}$ and $\hat{D}$) are created to facilitate the Kalman Gain computation (i.e., the next step of the PB-EKF) as well as the PB-MPC algorithm. These new output matrices are created using the elements of *y_volt,k_*. Considering the $\hat{D}$ matrix as an example, this is computed as follows using Algorithm 4:

(9) $$y_{volt,k}=f_{k}\left(j,\varphi_{e},\eta ,ocv\right)$$

**Algorithm 4: Compute the linearized matrices,**$\hat{C}$ and $\hat{D}$$clear\;\hat{D}$ $\hat{D}=D\left[17\right]*FR_{film}^{pos}-D\left[15\right]*FR_{film}^{neg}$ $\hat{D}+=D\left[6\right]$ $\hat{D}+=T_{K}*\varphi_{const}*\left(D\left[3\right]*1/c_{e,L}-D\left[0\right]*1/c_{e,0}\right)$ $ensure\;j_{0}^{neg}and\;j_{0}^{pos\;}are\geq minimum$ $R_{ct}^{neg}=T_{K}*cell_{RF}/j_{0}^{neg}$ $\hat{D}+=D\left[17\right]*R_{ct}^{pos}-D\left[15\right]*R_{ct}^{neg}$

The $\hat{C}$ matrix comprises a term that is derived from *OCV,* which is computed similarly to ΔOCV. After computing the $\hat{D}$ and $\hat{C}$, the former becomes a scalar and the latter becomes a vector, which in turn dramatically reduces the computation complexity of both PB-EKF and PB-MPC. Next, the Kalman Gain matrix (*L*) is computed using the following Algorithm 5, corresponding to the Equation (10) (i.e., Equation (e) in Figure 3):

(10) $$L=\frac{\sigma_{x,k}{\hat{C}}^{T}}{\sigma_{y}}$$

**Algorithm 5: Compute the Kalman Gain matrix, L**$clear\;\sigma_{x},\sigma_{y}$ $for\;i=1\;to\;N_{s}$ $\{for\;j=1\;to\;N_{s}$ $\left\{{\hat{C}}_{\sigma_{x}}\left[i\right]+=\hat{C}\left[j\right]*\sigma_{x}\left[j\right]\left[i\right]\right\}\}$ $for\;i=1\;to\;N_{s}$ $\{\sigma_{y}+={\hat{C}}_{\sigma_{x}}\left[i\right]*\hat{C}\left[i\right]$ $\sigma_{y}+=\hat{D}*\hat{D}*\sigma_{v}\}$ $for\;i=1\;to\;N_{s}$ $\left\{L\left[i\right]={\hat{C}}_{\sigma_{x}}\left[i\right]/\sigma_{y}\right\}$

In this case, $\sigma_{y}$ is the voltage error covariance, which is computed using the Equation (11) (corresponding to the Equation (f) in Figure 3); and $\sigma_{v}$ is the error in voltage measurement, which is a constant in this PB model.

(11) $$\sigma_{y}=\hat{C}\sigma_{x,k}{\hat{C}}^{T}+\hat{D}\sigma_{v}{\hat{D}}^{T}$$

After computing the Kalman Gain matrix (*L*), in Stage 6, the PB-EKF computes the state and error covariance measurement update using Algorithm 6 (corresponding to Equation (12), i.e., Equation (g) in Figure 3) and Algorithm 7 (corresponding to Equation (13), i.e., Equation (h) in Figure 3), respectively. The error covariance measurement update utilizes *L* and the innovation. In this case, the innovation is defined as the difference between the measured cell voltage and the output prediction. The state is computed using Algorithm 6, corresponding to Equation (12) as follows:

(12) $$x_{k+1}=x_{k}+L\left(v_{cell,k}-y_{volt,k}\right)$$

**Algorithm 6: Compute the state measurement update**$for\;i=1\;to\;N_{s}$ $\left\{x_{ekf,plus}\left[i\right]=x_{ekf}\left[i\right]+L\left[i\right]*innovation\right\}$

The error covariance measurement update is computed as follows using Equation (13):

(13) $$\sigma_{x,k+1}=\sigma_{x,k}+L\sigma_{y}L^{T}$$

The above parameter $\sigma_{y}$ is a scalar, and the algorithm can be simplified and written using Algorithm 7 as follows:**Algorithm 7: Compute the error covariance measurement update**$for\;i=1\;to\;N_{s}$ $\{for\;j=1\;to\;N_{s}$ $\left\{L_{\sigma_{y}L}\left[i\right]\left[j\right]={\hat{C}}_{\sigma_{x}}\left[i\right]*L\left[j\right]\right\}$ $for\;i=1\;to\;N_{s}$ $\{for\;j=1\;to\;N_{s}$ $\left\{\sigma_{x}\left[i\right]\left[j\right]=\sigma_{x}\left[i\right]\left[j\right]-L_{\sigma_{y}L}\left[i\right]\left[j\right]\right\}\}$

After computing the measurement update, the PB-EKF algorithm is technically completed. However, in order to support the PB-MPC algorithm as well as to support the SOC compensator, the PB-EKF repeats Stages 3 and 4 (i.e., Equation (14) corresponding to Equation (i) in Figure 3) to updates the SOC, non-linear corrections, prior to computing Stage 7 for final updates for the PB-MPC.

(14) $$y_{var,k+1}=Cx_{k+1}+Du_{k+1}$$

In Stage 7, the EKF computes the derivative OCV value, two separate $\hat{C}$ and $\hat{D}$ linearized output matrices for both the side reaction overpotential ($\varphi_{s-e}$), and the SOC using the $x_{k+1}$ state. Thus, this $x_{k+1}$ state becomes the current-state of the PB-MPC. 

In our software design, the overall PB-EKF algorithm iterates/loops through the SOC update and the non-linear corrections one more time before exiting the loop in step 4e. This software flow for this iterative process is presented in Algorithm 8 below (which is also based on the stages in Figure 3). The aforementioned repetition of Stages 3 and 4 (in Equation (14), i.e., Equation (i) in Figure 3) corresponds to the steps 4a to 4d. Our algorithm constructs reduce the code size for our embedded software architecture, leading to more efficient embedded design.
**Algorithm 8: Functional Flow of Extended Kalman Filter***1.* *Build A matrix**2.* *Compute the state time update, (x)**3.* *Compute the error covariance update, (σ)**4.* *Loop (for 1 to 2)**a.* *Compute the SOC update**b.* *Build C and D matrix**c.* *Calculate term s*$\varphi_{e}$*and*Δη*from Non-linear corrections**d.* *Calculate the predicted output voltage,*$(y_{volt,k)}$*e.* *If Loop = 2, break**f.* *Build*$\hat{C}$*and*$\hat{D}$*g.* *Compute the Kalman Gain matrix (L)**h.* *Compute the state measurement update, (x)**i.* *Compute the error covariance measurement update, (σ)**5.* *End Loop**6.* *Create terms needed for MPC formation and constraints**a.* *Calculate the updated Non-linear φ_s-e_**b.* *Compute the derivative of OCV at current state/soc**c.* *Create the C and D matrices for MPC SOC and φ_s-e_ output equations**7.* *End EKF*

The outputs of the PB-EKF are forwarded directly as the inputs of the PB-MPC, which is detailed in the next sub-section. 

### 3.2. MPC

The functional flow of our PB-MPC software is presented in Algorithm 9 as follows:
**Algorithm 9: Functional Flow of Physics-Based Model Predictive Control***1. Calculate next state for the state vector based on current state from EKF* *2. Calculate change increment for the state vector and control signals* *3. Compute E and F from the cost optimization forms* *4. Compute the unconstrained solution*$DU=E^{-1}F$ *5. Build M and γ based on*$G_{\varphi_{se}}$*and*$\Phi_{\varphi_{se}}$*from Equation (18)* *6. If M (DU) ≤ γ is true, END MPC, do nothing.* *7. Else execute Hildreth QP to provide constrained solution increment*$\Delta U_{k}\left[0\right]$ *8. Update control signal,*$i_{gref}^{*}=i_{gref}+\Delta U_{k}\left[0\right]$ *9. Final constraint check on*$i_{gref}^{*}$*to insure it stays within operating bounds* *10. Update SOC_mpc_ with SOC from EKF*

Although the PB-MPC has some overlapping functions, similar to the ones in traditional MPC ([2,4]), the PB-MPC often tracks the current to ensure safe operation, but does not concern itself with the SOC balancing or voltage regulation. In this case, the PB-MPC operates on the difference between the current-state and next-state, mainly due to the embedded integrator, whereas the PB-EKF operates on the amplitude of the control signals. The former (i.e., PB-MPC) requires configuring the traditional MPC algorithm. As a result, the PB-MPC computes the next-state using *A* and *B* matrices generated from the PB-EKF, and computes the difference between the states using Equation (15) as follows:

(15) $$x_{m,k+1}=Ax_{m,k}+Bu_{mpc,k},\;\mathrm{and}\;\Delta x_{m,k}=x_{m,k+1}-x_{m,k}$$

In Equation (15), from PB-EKF, $x_{m,k}$ = $x_{k+1}$. Considering the zero/non-zero *D* terms, the final state vector is defined using Equation (16):

(16) $$\chi_{k}=\left[\begin{array}{c} x_{mpc,k}\\ u_{mpc,k} \end{array}\right],\;\mathrm{where}\;x_{mpc,k}=\left[\frac{\Delta x_{m,k}}{y_{k}}\right]$$

Furthermore, the state-space and the output equations are presented as Equation (17) and Equation (18), respectively:

(17) $$\chi_{k+1}=\tilde{A}\chi_{k}+\tilde{B}\Delta u_{mpc,k+1}$$

(18) $$y_{k}=\tilde{C}\chi_{k}$$

where the state and output matrices are redefined as follows in Equation (19):

(19) $$\tilde{A}=\left[\begin{array}{cc} A & B\\ 0 & I \end{array}\right],\;\tilde{B}=\left[\begin{array}{c} 0\\ I \end{array}\right],\;\tilde{C}=\left[\begin{array}{cc} \hat{C} & \hat{D} \end{array}\right],\;\mathrm{and}\;\Delta u_{mpc,k+1}=\;u_{mpc,k+1}-u_{mpc,k}$$

In order to initialize our PB-MPC software architecture as stated in steps 1 and 2 of the above software functional flow (in Algorithm 9), we utilize the Algorithm 10 as follows:**Algorithm 10: MPC initialization of state, control signal, and SOC**$x_{mpc}=x_{ekf,plus}$ $for\;i=1\;to\;N_{s}$ $\;\{x_{mpc,plus}\left[i\right]=A\left[i\right]*x_{mpc}\left[i\right]+u_{k,mpc}\}$ $for\;i=1\;to\;N_{s}$ $\{\Delta \chi_{mpc}\left[i\right]=\;x_{mpc,plus}\left[i\right]-x_{mpc}\left[i\right]$ $\Delta \chi_{mpc}\left[N_{s}+1\right]=\varphi_{s-e}$ $\Delta u_{k+1,mpc}=u_{k+1,mpc}-u_{k,mpc}$ $SOC_{mpc}=SOC_{ekf}$

To compute step 3, it is imperative to understand that the cost function operates on the amplitude of the current, instead of the difference (or increment of change) between states. In order to facilitate this, the increment of change between states ($\Delta u_{mpc}$) is added to the previous control signal (*u*_0_) to create the amplitude (*i_g_*). This process is performed for each time step up to the prediction horizon. As in [2], *I_g_* is the vector of *i_g_* values, and Δ*U* is the vector of incremental change values, and *U*_0_ is the vector of the previous control signal. *I_g_* is represented by the following Equation (20):

(20) $$I_{g}=\Sigma \Delta U+U_{0}$$

where Σ is a lower triangular matrix. 

Then, in the vector form, the cost function is represented by Equation (21):

(21) $$J={\left(I_{g_{ref}}-I_{g}\right)}^{T}\left(I_{g_{ref}}-I_{g}\right)$$

The above Equation (21) is converted to the standard form as follows in Equation (22), to use on the Hildreth’s Quadratic programming (HQP) solution:

(22) $$J=\frac{1}{2}\Delta U^{T}E\Delta U+\Delta U^{T}F=\frac{1}{2}\Delta U^{T}\left(2\Sigma^{T}\Sigma \right)\Delta U+\Delta U^{T}(-2\Sigma^{T}\left(I_{g_{ref}-}U_{0}\right)$$

The PB-MPC uses the primal-dual approach (detailed in [14]) for optimizing the above cost function. The optimization is solved numerically by the HQP technique. This primal-dual approach incorporates the inequality constraints into the cost equation, and also uses *E* and *F* in the dual form as follows in Equation (23):

(23) $$\underset{\lambda \geq 0}{max}\left(-\frac{1}{2}\lambda^{T}P\lambda -\lambda^{T}K-\frac{1}{2}F^{T}E^{-1}F\right)$$

where $P=ME^{-1}M^{T}$ and $K=\gamma +ME^{-1}F=\gamma -M\left(DU\right)$.

After obtaining the above values, the PB-MPC computes step 4 (in Algorithm 9), which is the unconstrained solution *DU* as follows using Algorithm 11:**Algorithm 11: Compute the unconstrained solution for MPC**E=2∗Σ $F=-2*\Sigma *\Delta u_{k+1,mpc}$ DU=−F/E

In the PB-MPC, the main constraint is based on the side-reaction overpotential ($\varphi_{s-e}$) which is the primary cause of lithium plating and capacity loss as stated in [2]. The output vector for $\varphi_{s-e}$ is written as follows with Equation (24).

(24) $$Y_{\varphi_{se}}=G_{\varphi_{se}}\Delta x_{mpc,k}+\Phi_{\varphi_{se}}\Delta U_{k}$$

To compute step 5 (in Algorithm 9) of the PB-MPC, the constraint matrix *M* and vector *γ* are created from *G* and *Φ* from the above Equation (24). In case if the unconstrained solution *DU* violates any of the constraints in step 6, we use the HQP approach to compute the constrained control signs in step 7. From our previous work [4], it was observed that this iterative approach is suitable for embedded systems. However, the HQP approach does not always converge to an optimal solution, and instead might converge to a useable suboptimal solution [4]. This suboptimal solution computes a vector of Lagrange multipliers, one λ element at a time, where the λ element is a positive non-zero for an active constraint, and zero otherwise. The HQP converges when the previous λ and current λ are equal or within an acceptable variance. This λ vector is built using the *P* and *K* vectors detailed above. The summary of the HQP algorithm is presented below in Algorithm 12. The detailed description of this algorithm can be found in [4].
**Algorithm 12: Functional Flow of Hildreth’s Quadratic Programming solution for MPC, constrained solution***For iterations 1 to 40**1.* *Save λ_current_ → λ_previous_**2.* *LOOP1 to build λ, i = 0 to # elements in M or M_size_**a.* *w = 0;**b.* *LOOP2 to build λ, j starts at 0**i.* *w = w + P[i][j]∙λ[j]**ii.* *If j<Msize, repeat LOOP2 else GOTO 2.c**c.* *w = w + K[i]-P[i][i]∙λ[i]**d.* *λtest = -w/P[i][i]**e.* *if λtest< 0 then λ[i] = 0, else λ[i] = λtest**f.* *i<Msize repeat LOOP1 else GOTO 3**3.* *Check convergence**a.* *calculate the Euclidean length of previous λ**b.* *calculate the Euclidean length of current λ**c.* *Compare ratio to reference value**d.* *if converged is false GOTO 1, else GOTO 4.**4.* *Calculate new Δu**a.* *Start loop, j = 0 to j = M_size_**i.* Δ*u_c_ =* Δ*u_c_ +∙λ[j] ME^−1^[j]**b.* *GOTO 4.a if j<M_size_ else GOTO 4.c**c.* *DU =* Δ*u°-*Δ*u_c_**5.* *End*

The final steps (8, 9, and 10 in Algorithm 9) of the PB-MPC are to update the control signals if needed, then update the SOC in the PB-MPC, followed by updating the PB-EKF. Our software architecture then waits for the next interval.

## 4. Experimental Results and Analysis

We performed experiments to evaluate the feasibility and efficiency of our proposed embedded software architecture for the PB-EKF-MPC smart sensor. We compared our embedded software architecture with the baseline PB-EKF-MPC for BMS written in Matlab [2], to evaluate and validate the correctness and functionalities of our proposed design. Prior to the embedded software architecture, we created the software for PB-EKF-MPC in C and executed it on a desktop computer. These desktop results were compared and verified with the baseline Matlab results as well as with our embedded software results. Both the C and Matlab were executed on the same desktop computer comprising 16 GB Intel core i7 CPU running at 3.10 GHz. 

All our embedded software experiments were carried out on Xilinx V707 development board with Virtex-7 FPGA [15,16]. Our software modules were written in embedded C and executed on the 32-bit MicroBlaze soft-processor [17] running at 100 MHz on Virtex-7 FPGA, using Xilinx Vivado design tools [18,19]. The instantiation of the MicroBlaze soft-processor on the FPGA allowed the flexibility of configuring the embedded processor with varying memory sizes, and with different traits such as integrating a floating-point unit, etc., which in turn enabled utilizing only a single development platform/device to determine the minimum requirements of the microprocessor for our proposed embedded software architecture. For our embedded software, embedded C is used as the language of choice, since it can seamlessly be designed/executed on the MicroBlaze processor, and can also be ported to other embedded microprocessor with least modifications. For the embedded software, the fundamental operators are designed using single-precision floating-point (FP) units, whereas the baseline Matlab model [2] used double-precision FP operators. The experiments are based on the Urban Dynamometer Driving Schedule [13] profile for the *I_string_* input. The PB-EKF is initialized using a SOC of 0.6, whereas the PB-MPC is initialized with a SOC of 0.0. This PB-EKF-MPC smart sensor is designed to support a normal charging/discharging driving profile. 

### 4.1. Functional Verification

**Figure 4:**
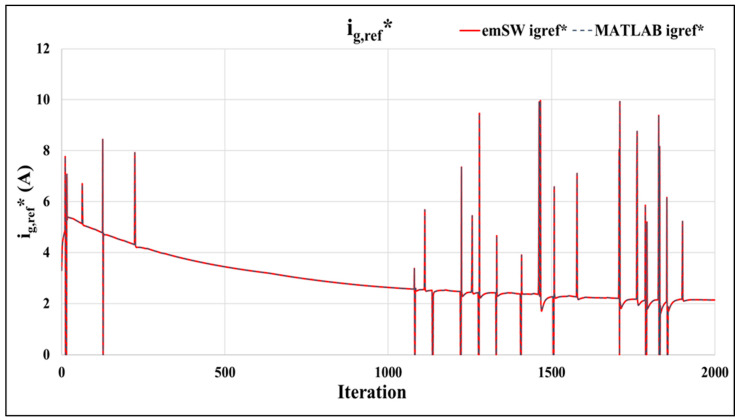
Control signals *I_gref_*: proposed embedded software vs. baseline Matlab.

It was imperative to ensure that our proposed embedded software architecture operated correctly. To facilitate this, we investigated three main outputs of the PB-EKF and PB-MPC, including the control signal ($i_{g_{ref}}^{*}$), side-reaction overpotential (*φ_s-e_*), and state of charge SOC. These three outputs could be utilized to determine the correct operation of our smart sensor. In this case, we compared our proposed embedded software for PB-EKF-MPC to the baseline Matlab PB-EKF-MPC in [2]. In order to execute our PB-EKF-MPC software design, it was essential to record the cell input current and cell reference voltage for the PB-EKF, as well as to record the input current $i_{g_{ref}}$ for the PB-MPC, at a standard UDDS driving cycle of 2000. Firstly, we examined the SOC, and the control signal *I_gref_*. As illustrated in Figure 4, our proposed embedded software results are quite close to the baseline Matlab results, except for the intermittent differences that start around the 1225th iterations and peak at the 1408th iteration. In this case, the standard deviation of the error is 512 μA, i.e., a percent error of −0.0000032%, which is negligible. 

**Figure 5:**
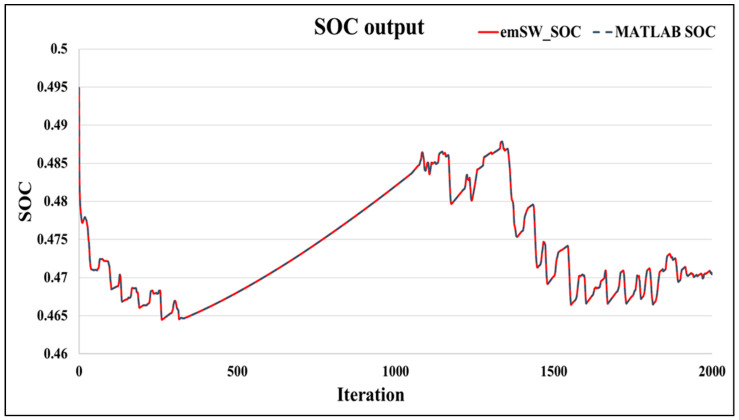
SOC output: proposed embedded software vs. baseline Matlab.

Secondly, we compared the SOC output from the PB-EKF, that is typically forwarded to the SOC compensator. Figure 5 illustrates the SOC output comparison between our proposed embedded software design and the baseline Matlab design. In this case, SOC output is a ratio, thus presented as a percentage. As shown in Figure 5, our proposed embedded software overlaps with the baseline Matlab design. The standard deviation of error between these two designs is 7.30014 × 10^−8^. This might be due to the single precision versus double precision FP operators used by our embedded software and Matlab software, respectively. 

**Figure 6:**
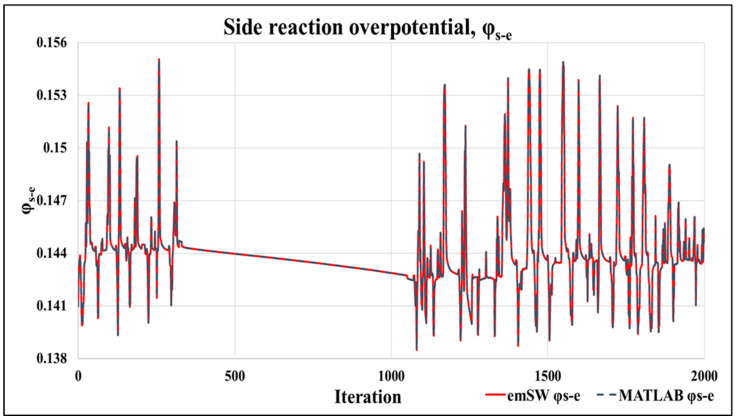
Side-reaction overpotential ($\varphi_{s-e}$): proposed embedded software vs. baseline Matlab.

Thirdly, we compared the side-reaction overpotential ($\varphi_{s-e}$), which is the key variable in the constraints for the proper operation of the PB-EKF-MPC as a smart sensor to prevent aging and performance degradation. Figure 6 illustrates the side-reaction overpotential comparison between our proposed embedded software design and the baseline Matlab design. As depicted, our proposed embedded software overlaps with the baseline Matlab. The standard deviation of error between these two designs is 3.26202 × 10^−8^, similar to the SOC output in Figure 5. The $\varphi_{s-e}$ does not exhibit any spikes. 

From the aforementioned results and analysis, it is evident that our proposed embedded software architecture achieves similar behavior and functionality as the Matlab design, and thus operates correctly. 

As stated in [2,20], the impact of aging is based on linking the side reaction overpotential to lithium plating, and the correlation between the lithium plating build up and the cell capacity decrease, which is also the useful life of the battery. The aforementioned experiments also prove that our system does not allow the side reaction overpotential to go into the negative by preventing *i_gref_* from exceeding the constraints. The overpotential graph (in Figure 6) and the *i_gref_* graph (in Figure 4) demonstrate that the BMS is successful in preventing the side reaction overpotential from entering a state that would increase the lithium plating, which in turn would decrease the cell capacity. Figure 6 also demonstrates that the overall design succeeds in maintaining $\varphi_{s-e}$> 0 to prevent lithium plating. By preventing lithium plating, the cell capacity of the battery is retained, thus extending battery life and usefulness. 

### 4.2. Performance Analysis: Execution Time, Speedup, and Code Size

For our embedded software architecture, the MicroBlaze processor is configured to have the maximum available on-chip cache memory of 128 KB, from which 64 KB is used for instruction cache and 64 KB is used for data cache. In order to execute our proposed embedded software architecture for PB-EKF-MPC on the MicroBlaze processor both efficiently and effectively, we set both the stack and heap sizes to 20 KB each. Table 1 presents the code size breakdown for our embedded software for PB-EKF-MPC, which demonstrates that our proposed embedded software can seamlessly be executed on a 32-bit embedded processor with limited memory (128 KB cache memory). It is imperative for the embedded software codes to be lean in order to be executed on the resource-constrained embedded devices. 

During the design phase, we integrated the AXI Timer [21] to measure the execution times of our embedded software designs. As our embedded software execution times, we obtained and reported the “worst-case time” to execute a single iteration of our embedded software PB-EKF-MPC smart sensor, in Table 2. This “worst-case time” incurred when it was absolutely necessary to execute the HQP algorithm due to a violation of a constraint by the unconstrained solution. Thus, as illustrated in Table 2, the “worst-case time” (in column 2) depends on the number of iteration counts utilized for the HQP algorithm (in column 1). In this case, the HQP iteration count is varied, since this count directly correlates with achieving an optimal solution. From Table 2, our highest worst-case time for the highest HQP iteration counts (i.e., 500) is 68.2 ms (row 1, column 2) to process our embedded software architecture for PB-EKF-MPC smart sensor. 

From our experimental results and analysis, it is also observed that the HQP algorithm is invoked only 3.7% of the time during the first 2000 iterations of our embedded software for the PB-EKF-MPC smart sensor, with an average execution time (i.e., average time per iteration) of 4.8 milliseconds (ms). However, in order to determine how many battery cells a typical single embedded microprocessor can support, we decided to use the worst-case time (as in Table 2) to ensure ample margin for good performance. 

As stated in [2], the standard sampling time (or interval) for battery cell is 1 s (1 s). In this case, only a portion of this 1 s interval can be utilized to execute our proposed embedded software PB-EKF-MPC smart sensor, since there are numerous other control algorithms needed to be executed during this 1 s interval. In Table 2, we present the number of battery cells that can be supported for 1, 0.5, 0.4, and 0.3 s intervals for varying HQP iterations, in columns 3, 4, 5, and 6, respectively. The long-term objective of the baseline Matlab algorithm in [2,3] was to utilize one microprocessor to support at most six battery cells, although the authors in [2,3] have not yet implemented/realized this algorithm physically on an embedded processor. Hence, if only one third (1/3rd) of the interval (i.e., 0.3 s) is available to execute our proposed embedded software PB-EKF-MPC smart sensor, the maximum HQP iteration count would be 300 to support six battery cells using a single microprocessor, in order to balance the performance with available resources.

We also analyze and report the speed-performance between our embedded software architecture and the baseline Matlab model, in Table 3. The execution times (in column 2 from Table 3) are based on the HQP iteration count of 500. The average time is taken from 1200 cycles. As illustrated, our embedded software still achieves an average execution time of 4.8 ms running at a very low clock frequency of 100 MHz on an embedded processor, while the baseline Matlab achieves an average execution time of 3.1 ms running at a much higher clock frequency of 3.1 GHz on a desktop computer. In this case, our embedded software is only 1.3 ms slower than the Matlab counterpart. This demonstrates that our proposed embedded software speedup is still substantial, considering our embedded software is only running at 100 MHz, versus the baseline Matlab model running at 3.1 GHz. 

The above experimental results and analyses illustrate that our proposed embedded software architecture for the PB-EKF-MPC smart sensor is a viable approach for battery cell management. 

### 4.3. Analysis of Existing Works on Embedded Software Designs for PB-EKF-MPC

We investigated the existing works on embedded software architectures for the PB-EKF-MPC in the published literature. Most of the existing works focused on Matlab modeling to verify and improve the physics-based (PB) approaches. In this paper, our research work is inspired by the existing Matlab model using the Discrete Realization Algorithm (DRA) approach in [2,3,6,10,11,12] from Trimboli and Plett’s group, which has done extensive work on lithium-ion battery cell management. 

In addition, we observed that the single partial model (SPM) approach was also popular. In [5], authors proposed an EKF that incorporates electrolyte dynamics into the SPM, to enhance this model with visibility into the electrochemical reactions. The SPM is further extended in [8] to develop a thermo-electrochemical single partial (SP) lithium-ion battery model and a novel moving horizon estimation approach. In [9], the authors used a thermo-electrochemical SPM for battery pack control with a sampling time of 40 s compared to 1 s as in our work. It should be noted that none of the aforementioned were embedded software designs. 

Our investigation revealed that there were a few PB-EKF-MPC software designs utilized for non-battery fields such as unmanned air [22] and underwater vehicle control systems. However, none of these were embedded software designs. For instance, in [7], the PB-EKF-MPC software tracking system was proposed for underwater vehicles, and was executed on a 16 GB i7 CPU running at 2.9 GHz with an average execution time of 50 ms. Furthermore, in [23], a moving-horizon-estimator-based MPC was proposed for a ground robot controller, which was executed on a CPU running at 1.2 GHz with an average execution time of 0.6 ms. 

From this investigation and to the best of our knowledge, no similar work is found in the published literature, and our proposed work is the first full embedded software architecture for the PB-EKF-MPC, specifically for battery cell control and monitoring. Our embedded software design is executed on the 32-bit embedded MicroBlaze processor running at 100 MHz with 128 KB memory, and still obtains an average execution time of 4.8 ms.

## 5. Conclusions and Future Work

In this paper, we introduced a novel, unique, and efficient embedded software architecture for a PB-EKF-MPC smart sensor for lithium-ion battery cell control, by addressing the computational complexity of PBM. Our embedded software codes were created in such a way as to be lean in order to execute on a 32-bit embedded microprocessor with a very limited memory of 128 KB. Our proposed embedded software architecture for the PB-EKF-MPC performed well within the 1 s update intervals of the BMS when running at only 100 MHz. Our embedded software architecture achieved an average execution time of 4.8 ms compared to 3.1 ms for its Matlab counterpart, which was still substantial considering our embedded software was only running at 100 MHz, whereas Matlab was running at 3.1 GHz. All our experimental results and analyses illustrated that our proposed embedded software architecture for the PB-EKF-MPC smart sensor is indeed a viable approach for battery cell management. 

Furthermore, with our proposed embedded software architecture for PB-EKF-MPC, we demonstrated that it was possible to support six battery cells (in a battery pack) using a single small-footprint embedded microprocessor, which was the long-term objective of the original baseline Matlab model in [2,3]. It should be noted that authors in [2,3] have not yet realized this algorithm on an embedded microprocessor. Our experiments also demonstrated that controlling the reference current prevented the side reaction overpotential from entering a negative state and thus blocking one of the causes of lithium plating from occurring. By preventing the lithium plating, the cell capacity of the battery is retained, thus extending the useful life of the battery. 

From our investigation on the existing works and to the best of our knowledge, we could not find any similar work in the published literature that provided a complete embedded software architecture for the PB-EKF-MPC as a smart sensor, specifically for battery cell monitoring and control, which highlights the uniqueness of our contribution. Our research work affirmed that a software-only embedded solution of a successful BMS approach comprising compute-intensive and highly accurate models/algorithms is still feasible on a small-scale embedded microprocessor running at 100 MHz with a very limited memory of 128 KB. Our unique embedded solution/approach provides the benefit of computationally complex elements of the PBM, EKF, and the MPC control algorithms to seamlessly fit into a resource-constrained environment; thus, our work opens up avenues to mobile and embedded applications, and asserts that the research work done and approaches proposed are not only theoretical but are achievable and applicable in real-world scenarios.

As future work, we are planning to create efficient embedded hardware architecture for the PB-EKF-MPC smart sensor. Our previous analysis [24] illustrated that Field-Programmable Gate Arrays (FPGAs) is one of the best avenues for creating hardware architectures on resource-constrained embedded devices. Previously, we introduced FPGA-based hardware architectures to support and accelerate several compute- and data-intensive applications, including data analytics [25,26], control systems [4,27,28], machine learning [29,30], and cybersecurity [31,32], specifically on embedded devices. We believe that customized and optimized FPGA-based hardware architectures/techniques would further improve the speed-performance of the PB-EKF-MPC smart sensor, while addressing the stringent requirements of embedded devices. 

For additional future work, we are planning to investigate and incorporate dynamic reconfiguration techniques [33,34] to create dynamic reconfigurable hardware architectures (similar to [35,36]) in order to further improve the smart and adaptability traits of the PB-EKF-MPC smart sensor.

Another potential future work is to explore and introduce parallel processing architectures (similar to [37,38]) for the PB-EKF-MPC to further enhance the speedup by considering the speed-space tradeoffs of the embedded devices. The PB-EKF-MPC algorithm comprises many computations that can be done in parallel. Hence, with FPGA-based hardware, we could certainly exploit parallelism in computations, including fine-grain, coarse-grain, data parallelism, etc. In this regard, we could also integrate multi-ported memory designs (such as [39,40]) to potential parallel processing hardware architectures, which would substantially elevate the performance. 

In addition, we are planning to explore the potential of expanding the physics based approach to monitor or control other aging chemical reactions. 

## Figures and Tables

**Table 1:** Our embedded software code size breakdown.

Executable Code (Bytes)	Data (Bytes)	Total Size (Bytes)	Total Size (Kilobytes)
60,352	41,023	101,616	99.23 KB

**Table 2:** Worst-case execution time and number of battery cells supported per sample time.

HQP Iteration Count	Worst Case Execution Time (Seconds)	Number of Battery Cells Supported per Available Control Interval Time			
		1.0 s Control Interval	0.5 s Control Interval	0.4 s Control Interval	0.3 s Control Interval
**500**	0.068213	14	7	5	4
400	0.055254	18	9	7	5
300	0.042236	23	11	9	7
250	0.035809	27	13	11	8
200	0.029254	34	17	13	10

**Table 3:** Performance comparison: embedded software architecture vs. baseline Matlab model.

Configuration	Average Execution Time (in ms)	Speedup over Embedded Software	Speedup over Baseline Matlab
Embedded software architecture (100 MHz)	4.811 ms		0.728
Baseline Matlab model (3.1 GHz)	3.503 ms	1.373	

## Data Availability

Not applicable.
